# The Immunological Therapeutic Strategies for Controlling Multiple Sclerosis: Considerations during the COVID-19 Pandemic

**DOI:** 10.3390/biom11091372

**Published:** 2021-09-17

**Authors:** Maryam Azimzadeh, Nora Möhn, Sajjad Ghane Ezabadi, Zahra Moghimi Esfandabadi, Alireza Soleimani, Elaheh Ranjbar, Maliheh Jahromi, Reihaneh Seyedebrahimi, Thomas Skripuletz, Farshad Moharrami Kasmaie

**Affiliations:** 1 Department of Medical Laboratory Sciences, Khomein University of Medical Sciences, Khomein, Iran; maryam.azimzade@khomeinums.ac.ir; 2 Department of Neurology, Hannover Medical School, 30625 Hannover, Germany; moehn.nora@mh-hannover.de; 3 Multiple Sclerosis Research Center, Neuroscience Institute, Tehran University of Medical Sciences, Tehran, Iran; s.ghane@ymail.com; 4 Faculty of Pharmacy, Tehran University of Medical Sciences, Tehran, Iran; zahra.moghimi.1378@gmail.com; 5 Department of Medical Laboratory Sciences, Faculty of Paramedicine, Rafsanjan University of Medical Sciences, Rafsanjan, Iran; soleimanialireza2021@gmail.com; 6 Department of Paramedical Sciences, Gonabad University of Medical Sciences, Gonabad, Iran; ranjbarelaheh2001@gmail.com; 7 Department of Anatomical Science, School of Medicine, Isfahan University of Medical Science, Isfahan, Iran; malihejahromi64@gmail.com; 8 Department of Anatomical Sciences, School of Medicine, Qom University of Medical Sciences, Qom, Iran; reihaneh.s.ebrahimi@gmail.com; 9 Department of Biology and Anatomical Sciences, School of Medicine, Shahid Beheshti University of Medical Sciences, Tehran, Iran; mkfarshad50.67@gmail.com

**Keywords:** multiple sclerosis (MS), COVID-19, disease-modifying therapies (DMTs)

## Abstract

A growing body of evidence initially suggested that patients with multiple sclerosis (MS) might be more susceptible to coronavirus disease 2019 (COVID-19). Moreover, it was speculated that patients with MS treated with immunosuppressive drugs might be at risk to develop a severe diseases course after infection with the severe acute respiratory syndrome coronavirus type 2 (SARS-CoV2). However, the recently published data have shown that MS patients do not have a higher risk for severe COVID-19. Although there is no indication that patients with MS and immunomodulatory/immunosuppressive therapy are generally at a higher risk of severe COVID-19, it is currently being emphasized that the hazards of poorly treated MS may outweigh the putative COVID-19 dangers. In this review, we discuss the challenges and considerations for MS patients in the COVID-19 pandemic.

## 1. Introduction

Multiple sclerosis (MS) is the most common inflammatory neurological disease in adults, especially between the ages of 20 and 40 and can potentially be a major cause of neurological disability in adulthood. Still, the pathomechanisms including basic questions related to the main causes of the disease have not been completely understood [1,2,3]. Because of the autoimmune-mediated inflammatory nature of the disease, treatment is based on immunomodulatory agents, including immunosuppressive therapies. At the onset of the COVID-19 pandemic, patients with MS encountered new challenges in both care delivery and clinical management as a result of various uncertainties.

The crucial concern for physicians of all specialties was identifying patients who might be at risk for a severe COVID-19 course. Initially, the potential risk of patients with MS could not be fully addressed. Furthermore, it was not clear if immunosuppressive agents might increase the risk of a severe acute respiratory syndrome coronavirus type 2 (SARS-CoV2) infection or the hazard to develop a severe disease course. The present paper aims to assess the different aspects of current therapeutic strategies and relevant challenges in the course and management of MS in times of the COVID-19 pandemic.

## 2. MS Disease Definition and Diagnosis

MS is characterized by inflammation in different areas of the central nervous system (CNS), including the optic nerve, brain parenchyma, brainstem, and spinal cord [4]. The simultaneous inflammation in different regions of the CNS is called dissemination in space (DIS). Dissemination in time (DIT) describes the recurrent inflammation of the CNS. Both criteria need to be fulfilled to diagnose MS—either regarding the clinical disease course or the pathological changes in magnetic resonance tomography (MRI) [5]. 

The diagnosis of MS is based on a combination of clinical, laboratory, and imaging findings by applying the McDonald criteria. The diagnostic criteria have evolved with the growth of technology and definitions were revised in order to be more accessible and usable for a larger segment of the population by maintaining sensitivity and specificity at the same time [6]. In 2017, the revision of these criteria determined changes based on evidence and restored the role of cerebrospinal fluid (CSF) oligoclonal bands [7]. Accordingly, MS can now be diagnosed more frequently at the time of a first clinical event by using the new criteria as compared to the 2010 McDonald criteria [8,9].

Standard definitions for the clinical course of MS are relapsing–remitting MS, primary progressive MS, and secondary progressive MS [10]. Most MS patients experience recurrent acute/subacute focal neurological deficits in different areas of the CNS. This common form of MS is known as relapsing–remitting MS (RRMS). About 35–50% of RRMS patients experience a steadily progressive neurological decline independent of prior inflammatory events, known as the “secondary progressive” phase (SPMS). In the absence of observable clinical relapses, about 15% of MS patients show a gradual, progressive decline from the beginning, a path known as “primary progressive” (PPMS). The definitions are based on the clinical course and do not provide information on the underlying pathophysiology of the disease. Although the course of the disease varies from mild to severe, the obtained data of relevant investigations examining the natural course of MS clearly show that the neurological damage worsens within 10–20 years in most untreated patients [11,12]. 

In simple words, environmental, genetic, and epigenetic factors play a role in MS pathogenesis and potentially interact with modifiable risk factors. Increasing evidence focused on identifying the prominent risk factors and their involvement in the development of MS [13]. Nowadays, there is a better understanding of risk factors contributing to the progression of the disease such as genetic (e.g., HLA DRB1 × 15:01), environmental (e.g., vitamin D or infectious mononucleosis, especially during puberty), and lifestyle (e.g., smoking) aspects. Ultimately, the disease is due to a dysregulation of the immune system with its crucial cellular protagonists, the microglia, activated macrophages, as well as B and T lymphocytes [14]. 

## 3. Concept of MS Immunopathogenesis

**Figure 1:**
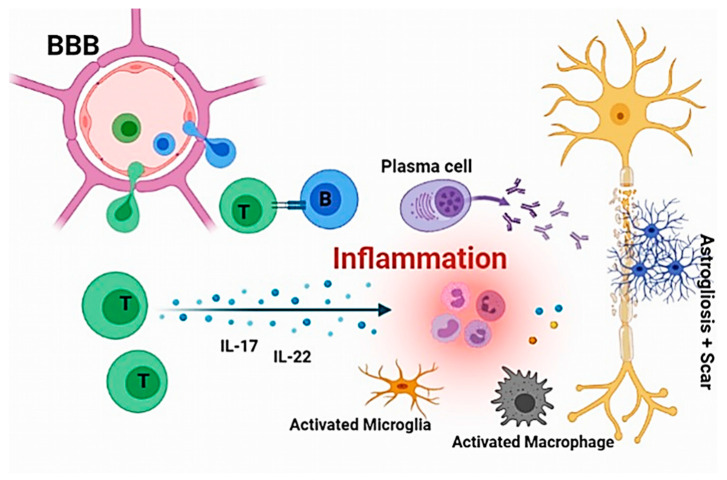
The initial immunopathogenesis of MS. A breakdown of peripheral T cell tolerance disrupts the blood–brain barrier (BBB) and leads to inflammation within the CNS resulting in the destruction of nerves.

Functionally, immunopathogenesis refers to the response of the immune system during the development of the disease. Despite speculation about the immune system’s specific role, the immunological failure to distinguish the self from the non-self, chronic CNS inflammation, and premature adaptive immunity changes are the predominant characteristics. Generally, the initial occurrence of MS disease is accompanied by a breakdown of peripheral T cell tolerance to myelin-associated antigens that causes the hallmark demyelination and neurodegeneration [15,16]. However, multiple hypotheses have been proposed regarding the immunopathological events involved in MS. For instance, it was stated that the immune cells invade the CNS, cause myelin sheath destruction, and inflammatory damage. In contrast, other evidence was noted that the primary abnormalities in the CNS itself could lead to inflammation and neuronal damage [17]. First of all, the humoral immunity components, glial cell function, and oxidative stress are the most important factors in the immunopathogenesis of MS due to their potent roles in inflammatory reactions. Biomarkers such as stimulatory molecules, inflammatory cytokine receptors, and microRNA alterations could intensify the Th2 cells activity versus Th1/Th17 activity, thereby reducing the function of regulatory T cells and increasing the risk of autoimmune diseases [18,19]. On the other hand, T-regulatory cell inefficiency limits the peripheral tolerance of B cells and leads to the proliferation of self-reactive B cell clones which, ultimately, induce the myelin sheath destruction by reactive Th1/Th17 cells. Subsequently, these reactions may lead to the disruption of the blood–brain barrier (BBB) and its destructive consequences (e.g., disruption and nerve damage) [20,21]. The other dominant inflammatory factors, including TGF-β and IL-21, increase the expression of IL-23R in Th cells [22,23]. The summarized initial mechanism of MS immunopathology is presented in Figure 1.

## 4. COVID-19 Pandemic Definitions

After the first discovered cases in Wuhan, China (December 2019), the novel coronavirus disease 2019 (COVID-19) was acknowledged as a global public health emergency. COVID-19 is caused by SARS-CoV-2, a novel RNA virus of the Coronaviridae family [24]. SARS-CoV2 is the successor to the 2002–2004 SARS outbreak (SARS-CoV-1) and the Middle East respiratory syndrome (MERS) (2012 to the present) and shares about 50–79% of its genetic sequence with the mentioned above coronaviruses, respectively [25,26]. The close contact through respiratory droplets, infected persons, contaminated objects/surfaces, and aerosols (i.e., in enclosed spaces indoors, crowded/inadequately ventilated spaces, and during aerosol-generating procedures) are the main transmission pathways of COVID-19. Although the viable virus detection could be accomplished up to 61 days post symptoms onset, virus shedding has been estimated to occur within the first 8 days from the onset of any symptoms (especially at the first 3 days) and takes place most commonly via the upper respiratory tract (URT) [27]. 

**Figure 2:**
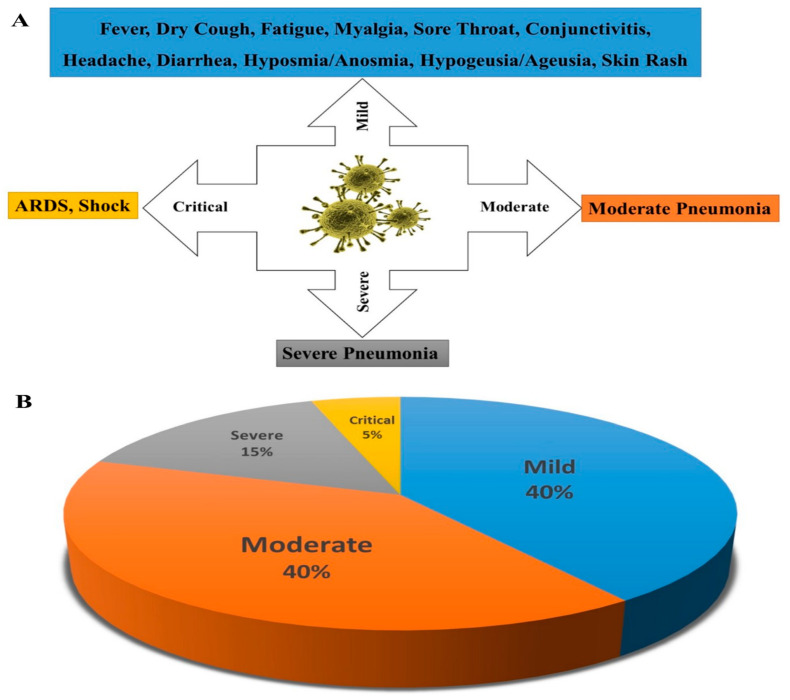
The clinical manifestations of symptomatic COVID-19 (**A**) and its prevalence (**B**).

COVID-19 may manifest as a symptomatic or asymptomatic disease. A recent meta-analysis estimated that about 17% of patient cases remain asymptomatic, while another systematic review reported an overall estimate of 31% [28]. It seems that the proportion of asymptomatic patients is controversial and should be better understood [28,29]. It was noted that fever, sore throat, and fatigue/myalgia were the most common symptoms in symptomatic patients [30]. However, congestion, rhinorrhea, and diarrhea were as well reported as less common symptoms [31]. In general, according to the latest version of the WHO COVID-19 Clinical management: living guidance, clinical manifestations can be classified into four stages (with their most prominent syndromes) (percentages may vary with variants and in vaccinated patients) (Figure 2):

1. Mild (fever, dry cough, fatigue, myalgia, sore throat, conjunctivitis, headache, diarrhea, hyposmia/anosmia, hypogeusia/ageusia, and skin rash): 40%,
2. Moderate (moderate pneumonia): 40%,
3. Severe (severe pneumonia): 15%
4. Critical (acute respiratory distress syndrome (ARDS) and/or shock): 5%.

The most frequently reported risk factors for a severe disease course and death are older age, cigarette smoking, and underlying diseases (e.g., diabetes mellitus, cardiac disease, hypertension, chronic lung disease, and cancer). The most commonly detected laboratory abnormalities are a reduced lymphocyte count, elevated C-reactive protein, and elevated lactate dehydrogenase [32]. Serology tests of IgM and IgG, nucleic acid assays, and gene sequencing have all been used to confirm the diagnosis of COVID-19. Moreover, it is frequently diagnosed via computed tomographic (CT) imaging; however, a chest CT scan may not be able to distinguish this disease from other viral causes of pneumonia [33,34].

## 5. Concept of COVID-19 Immunopathogenesis

**Figure 3:**
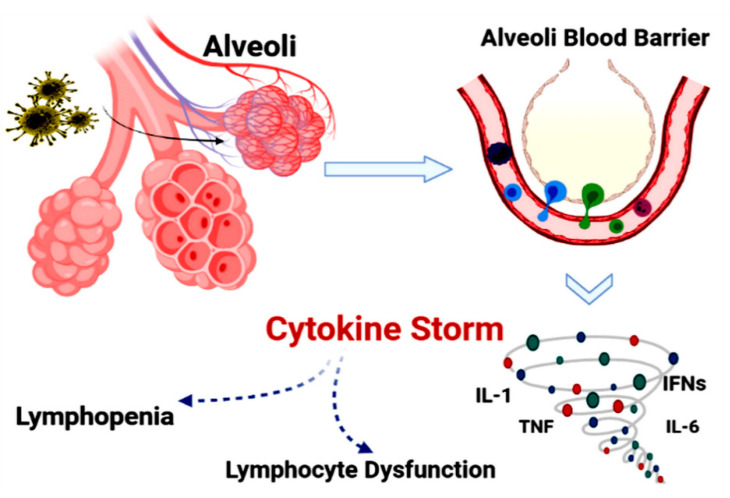
The immunopathogenesis of COVID-19. SARS-CoV-2 disrupts normal immune responses, leading to an impaired immune system and uncontrolled inflammatory responses (e.g., cytokine storm).

The feedback of the host immune system to COVID-19 plays a critical role in disease pathogenesis and clinical manifestations. Routinely, viruses are often identified post-infection by pattern recognition receptors (PRRs), such as the inflammasome sensor NLRP3, which trigger the production of interferons (IFNs) and inflammatory cytokines (e.g., IL-1, IL-6, and TNF) and initiate a local/systemic response to infection. In this process, innate and adaptive immune cells, including neutrophils, inflammatory myeloid cells, CD8^+^ T cells, and natural killer (NK) cells are recruited, activated, and differentiated. The cytotoxic activity of CD8^+^ T and NK cells, which allows virus-infected cells to be cleared, is crucial for infection resolution. Failure to clear virus-infected cells could lead to a hyper-inflammatory state known as macrophage activation syndrome (MAS) or “cytokine storm,” which induces lung injury (Figure 3). To address this, the findings of a thorough examination of gene expression data from COVID-19 patients’ blood, lungs, and airways suggested that COVID-19 pathogenesis is fueled by populations of myeloid-lineage cells in each compartment with highly inflammatory states. Especially, the lack of cytotoxic cells in the lungs indicates a scenario in which the delayed clearance of the virus enhances the myeloid cell activation to disease pathogenesis by producing inflammatory mediators. Moreover, the gene expression profiles could be applied to identify possible therapeutic targets for changing the treatment guidelines [35,36]. In brain cells, the ACE2 receptor (a key member in adsorption during SARS-CoV-2 infection) is expressed. However, its level and brain region expression appear to be limited. There is growing evidence that—in addition to systemic inflammation and thrombosis/emboli—the viruses’ direct action may cause neurological problems. The most common neurological symptoms linked with COVID-19 are headache and dizziness. However, in moderate and severe instances, neurological involvement appears to exacerbate. Infectious disorders such as COVID-19, which typically include a large number of asymptomatic patients, are dominated by mutant viruses that spread quickly. Because the probability of enhanced neurotoxicity based on the mutation cannot be ruled out, ongoing scientific and clinical studies are required [37,38]. As a result, B cells may be crucial in the fight against the SARS-CoV-2 virus. A greater knowledge of the protective responses of B cells during the infection would aid in the development of therapeutic therapies. However, there are various SARS-CoV2 clearance pathways which are not dependent on B cells, including the activation of CD8^+^ T cells or NK cells. Thus, the generation of anti-SARS-CoV-2 antibodies may not be required for an effective recovery from COVID-19 [36,37].

In severe forms of COVID-19, SARS-CoV-2 disrupts normal immune responses and leads to an impaired immune system with an uncontrolled inflammatory response. These patients exhibit lymphopenia, lymphocyte dysfunction, abnormalities of granulocytes and monocytes, increased production of cytokines, and increased total antibodies.

### 5.1. Lymphopenia, Lymphocyte Dysfunction

Lymphopenia is a key feature of COVID-19, especially in severe forms. Lymphopenic patients are more susceptible to microbial infections, which may intensify disease progression/severity. Increasing evidence indicated that lymphopenia could be served as a predictor of disease severity and prognosis of patients with COVID-19 [39,40,41,42]. In this regard, a reduction in the number of T, B, and NK cells was reported. The elevated exhaustion levels and dysfunction of T cells are other features that may predict severe COVID-19 [43]. As a result, several mechanisms may be responsible for lymphocyte depletion and dysfunction:

- The expression of ACE2 receptors on lymphocytes, especially T cells, may promote SARS-CoV-2 entry into them [44,45].
- An increase in cytokine levels (i.e., TNFα, IL-6, and IL-10) may promote the reduction and exhaustion of T cells [46].
- SARS-CoV-2 may destroy lymphatic organs (i.e., spleen and lymph nodes) [47].
- Lymphocyte proliferation may be inhibited by lactic acidemia, which is detected in severe COVID-19 [40,42].

### 5.2. Abnormalities of Granulocytes and Monocytes

The neutrophil/lymphocyte ratio is significantly higher in severe COVID-19 patients. Thus, it could be administered as an important indicator for an unfavorableCOVID-19 disease course. Moreover, eosinophils, basophils, and monocytes have reduced percentages in the severe phase of the disease. The likely mechanism behind the neutrophil upregulation in COVID-19 may have an association with lymphopenia, which predisposes to infection [48,49,50].

### 5.3. Increased Production of Cytokines 

Another key feature of severe COVID-19 is the increased inflammatory cytokine production, including IL-1β, IL-2, IL-6, IL-7, IL-8, IL-10, granulocyte-colony stimulating factor (G-CSF), granulocyte macrophage-colony stimulating factor (GM-CSF), interferon-inducible protein-10 (IP10), monocyte chemotactic protein 1 (MCP1), macrophage inflammation protein-1α, IFN-γ, and TNF-α [48,49,51]. This dramatic increase in cytokine levels over a short period is called a “cytokine storm” [39] in which the top elevated cytokines in severe cases are IL-1β, IL-6, and IL-10 [52,53]. The relevant mechanisms are supposed to be as follows:

- Following SARS-CoV-2 infection, GM-CSF secreted by the pathogenic T helper 1 cells activates CD14^+^CD16^+^ cells for more inflammation (mainly more production of IL-6) [54].
- Immune cell interaction in patients with COVID-19 is characterized by an increase in a subpopulation of CD14^+^ cells which may promote the level of IL-1β [55].
- The Th17 response was confirmed in patients with COVID-19. Studies have shown that Th17 cells recruit more immune cells to the infection sites, stimulating the cytokine cascades (e.g., IL-1β and IL-6) by producing IL-17 [56].
- Furthermore, eosinophils directly fight with RNA viruses, by releasing a large number of cytokines, among which IL-6 is a critical one to develop a cytokine storm in COVID-19 [57].

Finally, the cytokine storm may lead to viral sepsis, inflammatory-induced lung injury, ARDS, respiratory failure, shock, organ failure, and, potentially, death [39].

Additionally, in non-severe COVID-19—although significantly lower than severe ones—blood-cytokine levels (for example IL-1β, IL-1RA, IL-2R, IL-6, IL-7, IL-8, IL-9, IL-10, IFN-γ, TNF-α, G-CSF, GM-CSF, IP10, MCP1), can be increased [48,58,59].

### 5.4. Increased Antibodies

COVID-19 diagnosis is based on the detection of SARS-CoV-2-specific antibodies (IgM and IgG) alongside nucleic acid assays (NAAT/PCR). Zhang and colleagues showed that an increased IgG level is associated with disease severity [60]. Therefore, the IgG level could act as a simple marker to differentiate severe and non-severe cases. Moreover, a higher titer of total antibodies, which is significantly more quickly than that of IgM and IgG, was independently associated with a worse prognosis in COVID-19 [61]. Accordingly, B cell proliferation/activation in COVID-19 patients, especially in severe cases, is correlated with poor prognosis [53,62]. This could be explained by the antibody-dependent enhancement (ADE) of virus infection. ADE is a phenomenon in which preexisting sub-neutralizing antibodies promote virus entry and replication, such as what has been observed in the Ebola, Dengue, and MERS viruses [39].

## 6. MS and COVID-19

Immune system dysfunction is a crucial commonality between MS and COVID-19 which is caused by the incorrect activity of prominent immune cells, including T lymphocytes and their imbalance in the level of secreted anti/pro-inflammatory cytokines [39]. People suffering from autoimmune disorders, such as MS, were concerned about whether their immune system is capable of clearing SARS-CoV-2 effectively. MS patients need access to medical services for different purposes (i.e., routine visits, relapse management, DMT infusion sessions, rehabilitation services, MRI). In a previous study [63], the opinions of 360 neurologists from different countries were obtained on this subject. As a result, 98% reported facing COVID-19-related restrictions with telemedicine adopted to overcome this limited access to care. Likewise, 70% reported changes in using disease-modifying therapies (DMTs). It has been debated whether the COVID-19 pandemic would have a permanent influence on MS care or not [64]. These challenges could be classified into three categories:

- COVID-19 symptoms in patients with MS;
- Healthcare delivery to patients with MS;
- MS treatment and its safety considerations in COVID-19.

### 6.1. COVID-19 Symptoms in Patients with MS

In a recent systematic review, the most common symptoms of COVID-19 in patients with MS were as follows: fever (68.8%), cough (63.9%), fatigue/asthenia (51.2%), and dyspnea (39.5%) [65]. In total, only 5.3% of patients were asymptomatic. The total hospitalization and mortality rates in patients with MS and suspected/confirmed COVID-19 were 20.7% and 3.0%, respectively. In this regard, the highest hospitalization and mortality rates were in individuals with no DMTs (42.9% and 8.4%), followed by those treated with B-cell-depleting drugs (29.2% and 2.5%). Similar results were described in another systemic review of 873 published cases of MS and COVID-19 [66]. The mortality rates were higher in MS patients without DMTs as compared to MS patients on therapy. However, it should be noted that older patients with advanced stages of MS and severe cardiovascular comorbidities were generally not treated with DMTs due to risk–benefit assessments. It was stated that immunosuppressive or immunomodulatory therapies do not appear to be a substantial risk factor for a severe COVID-19 course. However, compared to other DMTs, patients receiving B-cell-depleting therapies presented higher hospitalization and mortality rates. Again, it should be noted that diseased patients receiving B-cell-depleting therapies were also at higher ages and presented a severe degree of disability. Notwithstanding, more established investigations are required [65,67]. 

The major risk factors for severe COVID-19 in patients with MS were older age, high disability levels, progressive course, obesity, and cardiovascular comorbidities. Accordingly, published data suggest that MS may not significantly increase the mortality rate of COVID-19. There was no significant difference between the severity and mortality of COVID-19 in patients with MS and the general population [65,68]. Bsteh and colleagues announced that the proportion of patients with MS at high risk of COVID-19 mortality is under 1% [69]. Moreover, a newly published analysis of the US electronic health records (IBM Explorys dataset and Biogen GSD) showed that patients with MS did not have a higher risk of severe COVID-19 and it was shown that comorbidities elevate the risk of infection [70]. Of note, the female to male ratio was 2.53:1 and the relapsing to progressive ratio was 4.75:1. The mean (SD) age, mean disease duration, and mean Expanded Disability Status Scale score were 44.91 (4.31) years, 12.46 (2.27) years, and 2.54 (0.81), respectively. At least one comorbidity was seen in 32.9% of patients [65].

### 6.2. Healthcare Delivery to Patients with MS

**Figure 4:**
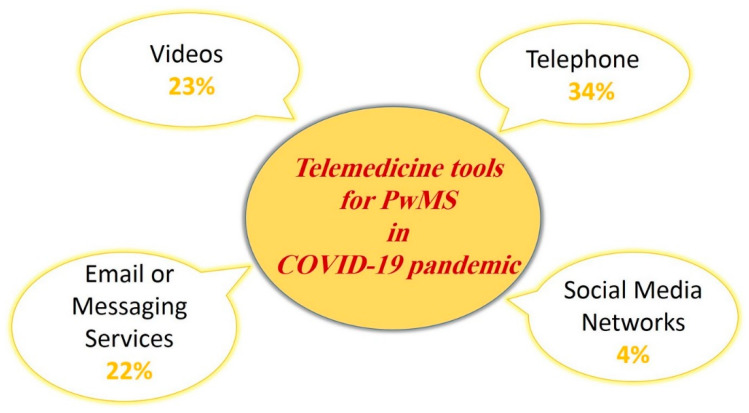
Utility of telemedicine as the care management tool for patients with MS (PwMS) in the COVID-19 era. The percentages were extracted from the ECTRIMS study [63].

Several studies demonstrated that COVID-19 has effects on healthcare delivery to patients with MS. The information varied to some extent in dependence of the reported countries, although there were some adaptations in triage, care delivery, and follow-up [63,71,72]. In an ECTRIMS study [63], 88% of neurologists confirmed the fact that the access to care for patients with MS had changed in the COVID-19 era. It was declared that to overcome the limited access, telemedicine was the main strategy (being used by 92% of respondents primarily or exclusively) chosen. The so-called telemedicine tools consisted of calls via telephone (34%), video (23%), and services such as email or messaging services (22%). The remaining 4% used social media networks (Figure 4) [73,74]. Despite the benefits of telemedicine, there is a concern about data privacy. In other words, using the different telemedicine tools may impact the processing of patient’s data. Therefore, the principles of personal data protection should be observed in this regard [75]. COVID-19 also affected the access to MRI (urgent evaluation: 58%; suspended/postponed: 17%; performed regulatory: 19%; other: 6%) and laboratory tests (postponed: 37%; regularly performed: 30%; urgent evaluation: 28%; suspended: 2%) together with clinical trials activity (suspended:38%; postponed: 32%; regularly performed: 30%) [63]. 

### 6.3. MS Treatment and Its Safety Considerations in COVID-19

#### 6.3.1. DMTs

Most importantly, the COVID-19 pandemic raised concerns about patients with autoimmune diseases receiving immunosuppressive or immunomodulatory drugs. The hypothesis that these patients might be at higher exposure to SARS-CoV-2 infection and its complications could have a significant impact on their treatment. Despite the lack of strong evidence on the DMT’s consequence on COVID-19, the treatment guidelines (national and international) were developed based on expert consensus and the known mechanisms of drug action. These guidelines recommended delaying or re-treatment when using high-efficacy and lymphocyte-depleting drugs [68,76,77,78].

The Dutch cohort of MS and COVID-19 patients did not detect any clear association between severe outcomes of COVID-19 and DMT use or lymphopenia in patients with MS. This agreed with three studies regarding treatment with rituximab, cladribine, and alemtuzumab, in which all patients recovered from COVID-19 despite having lymphopenia [79,80,81,82]. In MS patients under DMT’s with mild COVID-19, it was recommended to continue DMT’s. In severe cases, initially, it was recommended to delay highly efficient DMT’s and corticosteroids [83,84]. However, the evaluation of each patient should be based on physical context and the progression stage of the disease. Currently, there is a broad consensus that all DMTs should be continued. To reduce the risk of infection and relapses, patients with MS were recommended to be vaccinated against SARS-CoV-2. The available evidence related to DMT’s is limited in the COVID-19 pandemic. Thus, it is necessary to analyze the virus-specific immune responses post SARS-CoV-2 vaccination [85]. To date, relatively little research has been conducted on the effects of SARS-CoV-2 vaccination in MS patients, in which no increased risk of relapses was announced [86,87]. However, the type of vaccine and genetic predisposition could be variable factors to understand this issue and conclusions in this regard require further investigations [88,89].

Monoclonal antibodies which selectively deplete B cells have been shown to significantly reduce disease progression in relapsing–remitting MS patients [90]. In PPMS, B-cell depletion was shown to be favorable and ocrelizumab was the first medicine to be approved for this disorder. Despite recent significant breakthroughs in the understanding of B cells’ role in MS, there is still more to learn. The development of more effective and safe B-cell therapy is dependent on a better consideration of B-cell biology and more investigation on, as well as a better knowledge of, MS pathophysiology [91]. 

The data are inconsistent, as some studies mentioned a likely protective role for anti-CD20 monoclonal antibodies such as ocrelizumab and rituximab against COVID-19 [92,93,94]. On the other hand, it was suggested that these monoclonal antibodies may predispose patients with MS to COVID-19 and a critical case of COVID-19 infection and death post-treatment with rituximab was reported [82,95].

Therefore, initially, there was concern about the high-potent drugs, including sphingosine-1-phosphate modulators (S1P), B-cell-depleting agents, alemtuzumab, and cladribine, to potentially escalate the risk of severe COVID-19 disease course in patients with MS. Thus, cladribine and alemtuzumab were suggested to be contraindications to use [83,96]. In contrast to the national/international guidelines suggestions, most recent studies did not confirm the initial concerns. It was reported that even during treatment with highly effective therapeutics, including alemtuzumab, a severe COVID-19 course was not necessarily to be expected. One interesting case presented a young female MS patient with only a mild course of COVID-19 despite significant lymphopenia due to treatment with alemtuzumab which she received a few days before SARS-CoV-2 infection [82].

Initially, the general acknowledgment indicated that interferons and glatiramer acetate (GA) are safe and interferons could be even protective in severe COVID-19. Subsequently, it was implied that patients taking interferons or GA might have the lowest, and those receiving high-potent drugs such as anti-CD20 therapies might have the highest risk for severe COVID-19 [70]. Furthermore, Brownlee stated that “we are now ready to reinstitute the standard MS treatment protocols” since the risk of developing a disability from delayed initiation or re-treatment with a high-potent medication will be heavier than the risks of severe COVID-19 infection [97].

#### 6.3.2. Vitamin D

Vitamin D with its immune-modulating properties plays an important role in the pathogenesis of several autoimmune conditions (e.g., MS). Moreover, associations between low levels of vitamin D with ARDS, respiratory syncytial virus infection, and influenza have been described. In this regard, reducing the overproduction of cytokines could be a prophylactic therapy of exacerbated respiratory infection. On the other hand, supplementation of vitamin D deficiency in MS patients might be beneficial in controlling the inflammatory CNS processes. Accordingly, a daily administration of 2000–3000 international units (IU) vitamin D supplements was recommended by some authors for patients with MS [98,99,100,101,102,103].

#### 6.3.3. Vaccines

As the SARS-CoV-2 vaccines prevent severe progression, choosing an adequate vaccine is essential for patients with MS. It is important to consider several functional parameters as follows [86,104]:

- Safety:
  ○ Live-attenuated vaccines are contraindicated in patients with MS who are under highly effective DMTs.
  ○ Non-live COVID-19 vaccines are probably safe in patients with MS.
  ○ Most of the COVID-19 vaccines do not lead to demyelinating events. However, there are very rare reports mainly in those vaccinated with viral vector vaccines.
- Efficiency:
  ○ The efficacy of vaccines, in general, might be attenuated in patients treated with immunosuppressive therapies. Recently, it was shown that the mRNA-COVID-19 vaccine led to similar a SARS-CoV-2 IgG response in healthy subjects and untreated MS patients as well as in patients treated with cladribine. In contrast, a partial reduced humoral response was detected in patients treated with ocrelizumab and Fingolimod [105]. However, T-cell responses post vaccination were not investigated by the authors.

It may be better to coordinate the time of vaccination with DMTs prescription to achieve the optimum results from the vaccines. In general, COVID-19 vaccination is recommended for MS patients [105].

## 7. Conclusions

As the world was faced with the COVID-19 pandemic, which severely affects the immune system, healthcare staff had to overcome new challenges and burdens in both care delivery and clinical management of MS. Initially, immunosuppressive treatments were of great concern and, thus, data gathering on DMT safety were of great importance to help in treatment decisions. Furthermore, implementing new tools such as telemedicine were helpful in patients care management. Published data to date indicate that MS and the various immunotherapies do not significantly increase the mortality rate of COVID-19.

## Data Availability

The study did not report any data.
